# Hippocampal subfield volume and memory ability in healthy aging, subjective cognitive decline, and amnestic mild cognitive impairment

**DOI:** 10.1002/alz.095471

**Published:** 2025-01-09

**Authors:** Abi Heller, David Ellis, Caroline O Nester, Anna F Wilhelm, Nadia Pare, Laura Rabin, David E Warren

**Affiliations:** ^1^ University of Nebraska Medical Center, Elkhorn, NE USA; ^2^ University of Nebraska Medical Center, Omaha, NE USA; ^3^ The Graduate Center, CUNY, New York, NY USA; ^4^ Brooklyn College of the City University of New York, Brooklyn, NY USA

## Abstract

**Background:**

Subjective cognitive decline (SCD) is self‐reported experience of cognitive impairment, frequently memory, that sometimes precedes mild cognitive impairment (MCI) and Alzheimer’s disease (AD). While the hallmark memory deficits of AD are associated with pathological atrophy of the hippocampus, evidence that SCD is associated with changes in whole hippocampus volume is mixed. Subtle volumetric changes in the cytoarchitecturally distinct subfields of the hippocampus could be associated with SCD but have received limited attention. The current study aimed to leverage advances in high resolution brain imaging techniques to investigate whether differences in memory ability were associated with hippocampal subfield volumes in healthy aging, SCD and early AD.

**Method:**

91 older adults (mean age = 75.0 years) completed the Repeatable Battery for the Assessment of Neuropsychological Status (RBANS) delayed and immediate memory subtest, and were characterized as: cognitively unimpaired (CU, N = 29); SCD (N = 32); or amnestic MCI (aMCI, N = 30). Participants completed 3T MRI study that included a high‐resolution, in‐plane, turbo spin echo T2w image of the medial temporal lobe. Automatic Segmentation of Hippocampal Subfields (ASHS) software toolbox was utilized to segment the hippocampus into subiculum, CA‐1, CA‐2, CA‐3, and dentate gyrus (DG). Analysis of variance and post‐hoc comparisons of subfield volumes and memory index scores were compared between the CU, SCD and aMCI diagnostic groups.

**Result:**

ANOVA between groups revealed a significant difference in immediate *(F*(2,88) = 15.69, *p* < .001) and delayed (*F*(2,88) = 41.07, *p* < .001) memory index score, such that patients in the aMCI diagnosis group performed significantly less well than individuals in the CU or SCD groups. ANOVA between groups also revealed a significant difference in hippocampal subfield volumes between groups in subiculum (*F*(2,88) = 6.13, *p* < .01), CA‐1 (*F*(2,88) = 7.20, *p* < .01), and DG (*F*(2,88) = 6.13, *p* < .01), such that the aMCI group had significantly smaller volumes than the CU or SCD groups.

**Conclusion:**

The current study demonstrated significant differences in memory ability and hippocampal subfield volume between CU/SCD and aMCI patients. Characterizing changes hippocampal subfield volume in healthy aging and disease could lead to valuable insights on early disease markers associated with SCD, aMCI and eventual progression to AD.

